# TSH Levels in Subclinical Hypothyroidism in the 97.5th Percentile of the Population

**DOI:** 10.1155/2020/2698627

**Published:** 2020-06-13

**Authors:** Laura Pérez-Campos Mayoral, María Teresa Hernández-Huerta, Gabriel Mayoral-Andrade, Eduardo Pérez-Campos Mayoral, Edgar Zenteno, Ruth Martínez-Cruz, Héctor Martínez Ruíz, Margarito Martínez Cruz, Alma Dolores Pérez Santiago, Eduardo Pérez-Campos

**Affiliations:** ^1^Centro de Investigación Facultad de Medicina UNAM-UABJO, Facultad de Medicina y Cirugía, Universidad Autónoma Benito Juírez de Oaxaca, Oaxaca 68020, Mexico; ^2^CONACyT Facultad de Medicina y Cirugía, Universidad Autónoma Benito Juárez de Oaxaca, Oaxaca 68020, Mexico; ^3^Facultad de Medicina de la Universidad Nacional Autónoma de México, Ciudad de México 04510, Mexico; ^4^Tecnológico Nacional de México/IT de Oaxaca, Oaxaca 68030, Mexico; ^5^Laboratorio de Patología Clínica “Dr. Eduardo Pérez Ortega”, Oaxaca 68000, Mexico

## Abstract

The debate regarding the cutoff point in the treatment of patients with subclinical hypothyroidism (Shypo) is ongoing. Generally, two different groups are identified for treatment by levels of 10 and 20 mIU/L. Nevertheless, the question remains, “what cutoff point should be chosen?” We have written a selective nonsystematic review focused on the 97.5 percentile reference value reported in healthy subjects in a number of countries and observed important disparities, which partly show the challenge of identifying a single cutoff point for those patients needing medication. We identified studies of TSH on the natural history of subclinical hypothyroidism from population-based prospective cohort studies, which follow up patients for several years. The evolution of TSH levels in these patients is variable. Some cases of TSH may return to lower levels at different stages over the years, but others may not, possibly even developing into overt thyroid failure, also variable. We analyzed factors that may explain the normalization of serum TSH levels. In addition, we found that thorough population-based prospective cohort studies following up on TSH levels, thyroid antibodies, and ultrasonography are important in decisions made in the treatment of patients. However, the 97.5 percentile reference value varies in different countries; therefore, an international cutoff point for subclinical hypothyroidism cannot be recommended.

## 1. Introduction

Subclinical hypothyroidism (Shypo) is diagnosed when thyroid-stimulating hormone (TSH) is above the standard reference range of normal free thyroxine (FT4) [1]. Shypo is associated with coronary heart disease, heart failure, and increased cardiovascular mortality [2].

The reference ranges for laboratory tests are obtained by different methods, including Hoffmann [3], and commonly by the 95% confidence intervals of a population of healthy individuals. By definition, 5% of all “healthy” people's results will be outside the reference range and indicated as having “abnormal” values. With this method, 2.5% of healthy individuals may be identified as having high serum TSH values [4]. In addition, about 90% of all patients with Shypo have TSH levels of between 4 and 10 mIU/L (*μ*IU/mL) [5]. At the same time, some researchers maintain that a value of 10 mIU/mL is a reasonable threshold though the patients will be evaluated or treated [6].

We analyzed factors that could explain the normalization of TSH levels in patients with Shypo as well as TSH glycosylation, which may explain the increase in TSH half-life in these patients. Our goal is to analyze the cutoff points published to obtain a value that identifies patients with subclinical hypothyroidism. The importance of defining a cutoff point focuses on the fact that the use of levothyroxine in treatment may be associated with atrial fibrillation, osteoporosis, and most notably, increased mortality [7].

## 2. Materials and Methods

### 2.1. The 97.5 Percentile Reference Value Is Reported in Healthy Subjects

In order to compare prospective studies related to discrimination values [8] in the evolution of subjects with subclinical hypothyroidism to hypothyroidism requiring treatment, we reviewed the variations in TSH related to ethnic group and age in healthy subjects. First, we selected papers through a nonsystematic review, which reported healthy or thyroid-disease-free populations. Publications with less than 7 percentile ranks are reported, and those that did not match within the data tables and the ranges of the figures were excluded. The choice of criterion is the 97.5 percentile of TSH is reported in a table by age and gender. The works of Hollowell et al. [9], Vadiveloo et al. [10], Sriphrapradang et al. [11], and Sasso et al. [12] were selected and plotted.

One-way ANOVA followed by the Tukey multiple comparisons test was performed using GraphPad Prism, version 7.00, for Windows (GraphPad Software, La Jolla California, USA; http://www.graphpad.com).

### 2.2. Population-Based Prospective Cohort Studies

Next, also through a nonsystematic review, we made a comparison of the population-based prospective cohort study in order to identify TSH levels that predict the evolution of subclinical hypothyroidism to overt hypothyroidism. In this second search, the considered criteria included studies of patients who were followed up for one year or more and found to have a level of TSH associated with the probability of overt hypothyroidism.

We selected and analyzed the works of Fade et al. [13], Li et al. [14], Rosário et al. [15], and Somwaru et al. [16].

## 3. Results

**Figure 1:**
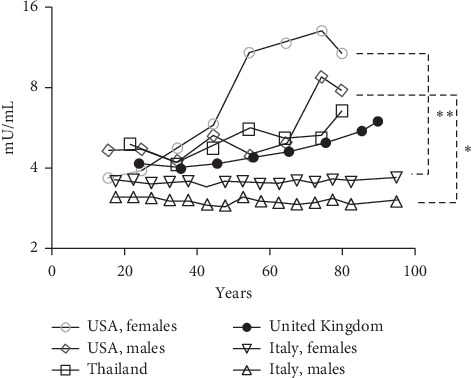
The 97.5th percentile TSH in healthy subject populations. There is a significant difference between males of Italy and USA (q 5,407) and between females of Italy and USA (q 9,320), using an ordinary one-way ANOVA and Tukey for multiple comparisons.

Variations in ethnic group, age, and gender are evident at around the 97.5 percentile reference value. Comparisons are shown in Figure 1. Serum TSH values in healthy subjects with no thyroid pathology vary in different populations and increase with age, as reported by the authors: (1) Hollowell et al. [9] in 533 subjects of USA from the National Health and Nutrition Examination Survey (NHANES) III used chemiluminescence immunometric assay (Nichols Institute Diagnostics, San Juan Capistrano, CA), with a working range of 0.01 to 50 mIU/L for this method. European-Americans, African-Americans, Mexican-Americans, and remaining ethnic groups were included in this study. (2) Vadiveloo et al. [10] used Roche Modular E170 (Roche Diagnostics, Lewes, East Sussex, United Kingdom), with a measuring range of 0.005 to 100 mIU/L (defined by the lower detection limit and the maximum of the master curve), in 62,368 subjects, the United Kingdom, Dundee. (3) Sriphrapradang et al. [11] used electrochemiluminescence immunoassay on a Cobas e411 analyzer (Roche Diagnostics, Mannheim, Germany), with a measuring range of 0.005 to 100 mIU/L, in 1947 subjects in four main regions of Thailand, i.e., North, Northeast, Central, and South. (4) Sasso et al. [12] used a Cobas e801 analyzer (Roche Diagnostics GmbH, Germany), with the limit of detection (LoD) of 0.005 mIU/L (Measuring Range 0.005 to 100 mIU/L), in 22602 subjects in Palermo, Italy. Figure 1 shows the 97.5 percentile of these populations. It is noteworthy that US subjects have higher levels of TSH than subjects in Italy. The difference in the results of Hollowell et al. [9] in the US population could be due to the fact that the method used has less sensitivity. However, the difference between the populations of Thailand, the United Kingdom, and Italy is remarkable, where they use similar methods.

With regard to the second objective, that of identifying TSH levels to predict the evolution of subclinical hypothyroidism, the results are compared as shown in Table 1.

The definitions of Shypo in different studies are not consistent, although a TSH cutoff point of 10 mIU/L has been used in all. The proportion of patients with Shypo that evolves into hypothyroidism is variable. It ranges from 2% in Somwaru et al. [16] to 19% in Fade et al. [13] studies. The normalization also varies from 5.5% in Fade et al. to 48% in Somwaru et al. studies and depends in part on the time range in which it is analyzed. The four studies agree that thyroid antibodies are a factor in progression. The level of TSH associated with an increased probability of overt hypothyroidism is relatively variable, with a range of 7 to 10 mIU/L.

## 4. Discussion

Although there is evidence to indicate that TSH levels vary in different ethnic groups, there are no comparisons between countries. In apparently healthy individuals, it has been shown that there are differences between African-Americans and European-Americans in the USA [17]. Also, in Bernalillo County (Albuquerque), New Mexico, Shypo is more common in women than in men and in non-Hispanic women than Hispanic women [18].

The major difference between US females and Italian females and US males and Italian males cannot be explained solely by genetic diversity or the differences between ethnic groups in the USA. The work of Murray et al. [19] identifies other disparities in factors such as income and basic health-care access. Moreover, in Italy, as in many other countries, something similar occurs with variations in languages spoken [20]. All these factors included in the exposome, in addition to the epigenome [21], may explain not just population variations of the disease but also differences in the analytes of healthy subjects, such as TSH levels.

In the debate about the upper limit for the TSH range of reference for Shypo, a limit of 4.4 to 5.0 mIU/L is generally accepted. However, there are groups of euthyroid individuals who have 2.5 mIU/L, depending on their iodine consumption; for example, having a previous iodized area with 2.12 mIU/L [22] could be a risk in overdiagnosis of Shypo, especially in adults.

Some interesting studies suggest an alternative cutoff based on the development of the disease; however, variations in TSH in a healthy population are too extensive for it to be obtained. In our review, we selected publications focusing on Shypo patients and found significant differences in their results. For example, in those that evolve to the overt hypothyroidism range of 2% to 19%, the persistence of Shypo ranges from 44% to 58%, and between 5% and 49% of patients are diagnosed as euthyroid. Also, an important difference in the presence of antibodies to TPO was reported by Rosário et al. [15] who do not consider this. In addition, Fade et al. [13] found TSH greater than 10 mIU/L in 81% of patients.

The huge difference in normalization between Fade et al. compared with Li et al. and Somwaru et al. could be explained in part by the difference in follow-up time, which was done just one year in the first study; in the other two, it was three and four years, respectively. Another notable difference is the sensitivity of the method used; in the study by Fade et al. [13], it is only 0.1 mIU/L, whereas in the studies by Li et al. [14], Rosário et al. [15], and Somwaru et al. [16], it is very similar 0.004 or 0.005 mIU/L. Focus on level, in Somwaru et al. report [16], an increase in TSH normalization was observed in patients with less than 6.9 mIU/L TSH and without thyroid antibodies.

Moreover, glycosylation on TSH modulates secretion, stability, bioactivity, metabolic clearance, and recognition by its receptor. Differences in sialylated, fucosylated, or sulfated subunits have shown between TSH subunits [23]. In hypothyroidism, increased sialylation and decreased sulfatation of TSH are observed. In addition, TRH-released TSH in subclinical hypothyroidism has less core fucose residues than TSH euthyroid subjects [24]. Highly sialylated circulating TSH isoforms escape hepatic clearance, and its metabolic clearance rate results in impaired intrinsic bioactivity and prolonged half-life [25, 26]. This could mean that higher levels of TSH occur in patients with subclinical hypothyroidism.

Regarding the relationship between TSH and anti-TPO, evidence can be found in the NHANES III, which shows that the age-related TSH levels may be independent of the level of thyroid antibodies [27].

In addition, TSH serum may be slightly higher in nonthyroid-related pathologies such as the following: morbid obesity, autoimmune disease, medications that cause hypothyroidism—such as amiodarone, lithium, interferon, antidopaminergics [1], bexarotene, corticosteroids, anti-CTLA4, and anti-PD-1 [28, 29]—all of which may modify thyroid tests, and also by interference from antibodies such as heterophils [30]. On the other hand, variable TSH levels could be related to iodine intake, smoking, ethnicity, and 64% by heritability [31].

A number of different researchers have suggested a criterion for treatment but only when there are signs and if the TSH level is >10 mIU/L or >20 mIU/L [14]. In subjects of 60 years and older, some of the signs for Shypo are not as clearly diagnosed as in those under 60 years [32]. TSH values may increase with age, which should lead to a more cautious interpretation of TSH in subjects aged over 60. It is noticeable that patients followed up with TPO antibodies and ultrasonography leads to a better diagnosis. Using ultrasonography, it is possible to identify autoimmune thyroiditis, when diffuse hypo-echogenicity is found compared with muscles [15].

With regard to the work of the Gourmelon group [27], it follows that signs such as periorbital oedema, slow movement, and thick skin are more specific to Shypo, although these have low sensitivity. Therefore, these authors suggest the evaluation of bioactive TSH, in particular, the ratio between bioactivity (B) and immunoreactivity (I), B/I can be useful [33]. To quote Mammen [34], “Additional research is needed to find novel ways to differentiate thyroid disease,” particularly in the elderly.

## 5. Conclusions

In conclusion, despite the evidence of a link between TSH levels and a greater probability of overt hypothyroidism, such as those indicating 7 to 10 mIU/L, it is not possible to define a single cutoff point. This is due to the large differences in levels between countries and ages (as shown in Figure 1), which, when combined with lifestyle, generates an impact on quality of life. However, it is clear that careful clinical analysis, together with the identification of markers such as TSH thyroid antibodies and ultrasonography, indicated in population-based prospective cohort studies, could help the clinician decide whether or not to give treatment in cases of subclinical hypothyroidism.

## Figures and Tables

**Table 1:** TSH in the natural history of subclinical hypothyroidism.

First author, year (ref.)	Fade, 1991 [13]	Somwaru, 2012 [16]	Rosário, 2016 [15]	Li, 2017 [14]
No. of patients; gender; and country	1193 over 60 yrs old; women (*n* = 700) and men (*n* = 510); and Birmingham, United Kingdom	459, at least 65 yrs old; women (*n* = 282) and men (*n* = 177); and USA communities	241 individuals of 20–71 yrs; women (*n* = 241); and Minas Gerais, Brazil	505 patients of 40 yrs or older; women (*n* = 168) and men (*n* = 138); and Shandong Province, China

Definition	Without being explicit, they define Shypo as abnormal TSH results, but normal free thyroid hormone (FT4)-TSH shows a positively skewed distribution with a reference limit of 0.5 to 5 mIU/L	Shypo was defined as having TSH of 4.5–19.9 mIU/L with a normal FT4	Shypo was defined as TSH being persistently >10 mIU/L, confirmed at an interval of 12 weeks	Shypo was defined by elevated TSH (mild TSH ≤10 mIU/L or severe TSH >10 mIU/L), with normal free thyroxine (FT4)

Limit of detection (LoD); TSH assay and method	0.1 mIU/L; MAIA-clone method (Serono Diagnostics)	0.005 mIU/L; Elecsys 2010 analyzer (Roche Diagnostics, Indianapolis, IN)	Assay range is 0.004–75 mIU/L; Immulite 2000 (Diagnostic Products Corporation)	Measuring range 0.005–100 mIU/L; Cobas E601 (Roche, Basel, Switzerland)

Follow-up of patients (years)	1	4	5	3

TSH (mIU/L) associated with an increased probability of overt hypothyroidism	10	10	8	7

Antithyroid antibodies and other factors related to progression to overt hypothyroidism	Progression to overt hypothyroidism was common in patients with antithyroid antibodies. 46% of patients with over 5 mIU/L TSH had antithyroid antibodies. 81% of patients with over 10 mIU/L TSH had antithyroid antibodies	Antithyroid peroxidase (anti-TPO) status. 35% of Shypo patients were TPOAb positive		Patients with total cholesterol >240.0 mg/dL had a greater risk of developing Shypo. TPOAb >34 IU/mL at higher risk of developing Shypo

Frequency of clinical and laboratory changes	The prevalence of abnormal TSH in patients was 13.8%. An inverse correlation was seen between TSH and FT4 in patients with TSH >5 mIU/L. High TSH values continued in 76.7% of patients for 12 months	69% with Shypo had TSH between 4.5 and 6.9 mIU/L. 56% of patients had persistent Shypo. 2%, at yr 2, developing overtly hypothyroid. 32%, at yr 4, were again Shypo	58.1% of patients had persistent Shypo. 19% of patients developed overt hypothyroidism	43.8% of patients had persistent Shypo. 3.4% of patients developed overt hypothyroidism

Frequency of TSH normalization	5% of patients had normalized TSH in 12 months	35% of patients had normalization of serum TSH at yr 2. 48% of patients who had reverted TSH remained euthyroid at yr 4. 45% of patients who had not reverted TSH had TPOAb at yr 4; 8% of patients had normalization in all samples at yrs 2 and 4	22.8% of patients had normalization of serum TSH at yr 5; and 51.5% of these were anti-TPO-negative patients	49.7% of patients had normalization of TSH serum

## References

[1] Biondi B., Cooper D. S. (2008). The clinical significance of subclinical thyroid dysfunction. *Endocrine Reviews*.

[2] Floriani C., Gencer B., Collet T.-H., Rodondi N. (2018). Subclinical thyroid dysfunction and cardiovascular diseases: 2016 update. *European Heart Journal*.

[3] Katayev A., Balciza C., Seccombe D. W. (2010). Establishing reference intervals for clinical laboratory test results: is there a better way?. *American Journal of Clinical Pathology*.

[4] Fatourechi V. (2007). Upper limit of normal serum thyroid-stimulating hormone: a moving and now an aging target?. *The Journal of Clinical Endocrinology & Metabolism*.

[5] Bekkering G. E., Agoritsas T., Lytvyn L. (2019). Thyroid hormones treatment for subclinical hypothyroidism: a clinical practice guideline. *BMJ*.

[6] Duntas L. H., Yen P. M. (2019). Diagnosis and treatment of hypothyroidism in the elderly. *Endocrine*.

[7] Grossman A., Feldhamer I., Meyerovitch J. (2018). Treatment with levothyroxin in subclinical hypothyroidism is associated with increased mortality in the elderly. *European Journal of Internal Medicine*.

[8] Sunderman F. W. (1975). Current concepts of “normal values,” “reference values,” and “discrimination values,” in clinical chemistry. *Clinical Chemistry*.

[9] Hollowell J. G., Staehling N. W., Flanders W. D. (2002). Serum TSH, T_4_, and thyroid antibodies in the United States population (1988 to 1994): national health and nutrition examination survey (NHANES III). *The Journal of Clinical Endocrinology & Metabolism*.

[10] Vadiveloo T., Donnan P. T., Murphy M. J., Leese G. P. (2013). Age– and gender–specific TSH reference intervals in people with no obvious thyroid disease in Tayside, Scotland: the Thyroid Epidemiology, Audit, and Research Study (TEARS). *The Journal of Clinical Endocrinology & Metabolism*.

[11] Sriphrapradang C., Pavarangkoon S., Jongjaroenprasert W. (2014). Reference ranges of serum TSH, FT4 and thyroid autoantibodies in the Thai population: the national health examination survey. *Clinical Endocrinology*.

[12] Sasso B. L., Vidali M., Scazzone C., Agnello L., Ciaccio M. (2019). Reference interval by the indirect approach of serum thyrotropin (TSH) in a mediterranean adult population and the association with age and gender. *Clinical Chemistry and Laboratory Medicine*.

[13] Fade J. V., Franklyn J. A., Cross K. W., Jones S. C., Sheppard M. C. (1991). Prevalence and follow-up of abnormal thyrotrophin (TSH) concentrations in the elderly in the United Kingdom. *Clinical Endocrinology*.

[14] Li X., Zhen D., Zhao M. (2017). Natural history of mild subclinical hypothyroidism in a middle-aged and elderly Chinese population: a prospective study. *Endocrine Journal*.

[15] Rosário P. W. S., Carvalho M., Calsolari M. R. (2016). Natural history of subclinical hypothyroidism with TSH ≤10 mIU/l: a prospective study. *Clinical Endocrinology*.

[16] Somwaru L. L., Rariy C. M., Arnold A. M., Cappola A. R. (2012). The natural history of subclinical hypothyroidism in the elderly: the cardiovascular health study. *The Journal of Clinical Endocrinology & Metabolism*.

[17] Schectman J. M., Kallenberg G. A., Hirsch R. P., Shumacher R. J. (1991). Report of an association between race and thyroid stimulating hormone level. *American Journal of Public Health*.

[18] Lindeman R. D., Schade D. S., LaRue A. (1999). Subclinical hypothyroidism in a biethnic, urban community,. *Journal of the American Geriatrics Society*.

[19] Murray C. J. L., Kulkarni S. C., Michaud C. (2006). Eight Americas: investigating mortality disparities across races, counties, and race-counties in the United States. *PLoS Medicine*.

[20] Saenz R., Rodriguez N., Embrick D. G. (2015). The international handbook of the demography of race and ethnicity. *International Handbooks of Population*.

[21] Siu C., Wiseman S., Gakkhar S. (2017). Characterization of the human thyroid epigenome. *Journal of Endocrinology*.

[22] Völzke H., Alte D., Kohlmann T. (2005). Reference intervals of serum thyroid function tests in a previously iodine-deficient area. *Thyroid*.

[23] Ząbczyńska M., Kozłowska K., Pocheć E. (2018). Glycosylation in the thyroid gland: vital aspects of glycoprotein function in thyrocyte physiology and thyroid disorders. *International Journal of Molecular Science*.

[24] Schaaf L., Trojan J., Helton T. E., Usadel K. H., Magner J. A. (1995). Serum thyrotropin (TSH) heterogeneity in euthyroid subjects and patients with subclinical hypothyroidism: the core fucose content of TSH-releasing hormone-released TSH is altered, but not the net charge of TSH. *Journal of Endocrinology*.

[25] Persani L., Borgato S., Romoli R., Asteria C., Pizzocaro A., Beck-Peccoz P. (1998). Changes in the degree of sialylation of carbohydrate chains modify the biological properties of circulating thyrotropin isoforms in various physiological and pathological states. *The Journal of Clinical Endocrinology & Metabolism*.

[26] Constant R. B., Weintraub B. D. (1986). Differences in the metabolic clearance of pituitary and serum thyrotropin (TSH) derived from euthyroid and hypothyroid rats: effects of chemical deglycosylation of pituitary TSH. *Endocrinology*.

[27] Surks M. I., Hollowell J. G. (2007). Age-specific distribution of serum thyrotropin and antithyroid antibodies in the US population: implications for the prevalence of subclinical hypothyroidism. *The Journal of Clinical Endocrinology & Metabolism*.

[28] Rizzo L. F. L., Mana D. L., Serra H. A. (2017). Drug-induced hypothyroidism. *Medicina (Buenos Aires)*.

[29] Stockigt J. R., Lim C.-F. (2009). Medications that distort in vitro tests of thyroid function, with particular reference to estimates of serum free thyroxine. *Best Practice & Research Clinical Endocrinology & Metabolism*.

[30] Chin K. P., Pin Y. C. (2008). Heterophile antibody interference with thyroid assay. *Internal Medicine*.

[31] Hansen P. S., Brix T. H., Sørensen T. I. A., Kyvik K. O., Hegedüs L. (2004). Major genetic influence on the regulation of the pituitary-thyroid axis: a study of healthy Danish twins. *The Journal of Clinical Endocrinology & Metabolism*.

[32] Gourmelon R., Donadio-Andréi S., Chikh K. (2019). Subclinical hypothyroidism: is it really subclinical with aging?. *Aging and disease*.

[33] Beck-Peccoz P., Persani L. (1994). Variable biological activity of thyroid-stimulating hormone. *European Journal of Endocrinology*.

[34] Mammen J. S. (2019). Interpreting elevated TSH in older adults. *Current Opinion in Endocrine and Metabolic Research*.

